# A Hypofractionated Radiotherapy Schedule with a Simultaneous Integrated Boost for Breast Cancer: Outcomes including Late Toxicity and Health Quality

**DOI:** 10.3390/medicina59040675

**Published:** 2023-03-29

**Authors:** Zuleyha Akgun, Aydin Cakir, Esra Sağlam, Sertac Demirel, Abdullah Igci, Serkan Keskin

**Affiliations:** 1 Department of Radiation Oncology, Memorial Sisli Hospital, Kaptan Paşa, Kaptan Paşa Mahallesi, Piyale Paşa Bulvari, Istanbul 34384, Turkey; 2 Vocational School, Department of Radiology, Istanbul Bilgi University, Sisli, Istanbul 34387, Turkey; 3 Department of General Surgery, Memorial Sisli Hospital, Kaptan Paşa, Kaptan Paşa Mahallesi, Piyale Paşa Bulvari, Istanbul 34384, Turkey; 4 School of Medicine, Department of General Surgery, Istanbul University, Istanbul 34452, Turkey; 5 Department of Medical Oncology, Memorial Sisli Hospital, Kaptan Paşa, Kaptan Paşa Mahallesi, Piyale Paşa Bulvari, Istanbul 34384, Turkey

**Keywords:** breast cancer, SIB, hypofractionation, hybrid radiotherapy, late toxicity

## Abstract

*Introduction*: This study aimed to evaluate the long-term adverse effects on the physical appearance and overall well-being of breast cancer patients who receive hypofractionated radiotherapy as whole breast and simultaneous integrated boost (SIB) treatment, utilizing intensive modulated radiotherapy (IMRT), volumetric modulated arc therapy (VMAT), or a hybrid therapy approach. *Material/Methods*: This investigation involved administering hypofractionated SIB-VMAT therapy to individuals diagnosed with early-stage breast cancer. Treatment was carried out over a three-week period in which a total dose of 48.06 Gy was given to the entire breast and 54 Gy was given to the tumor bed. Data on skin toxicity and cosmetic outcomes were analyzed both during the acute phase and during the three-month and five-year follow-up periods after treatment. *Results*: A total of 125 patients treated between December 2014 and December 2016 were included in the study. The data of these patients with at least 5 years of follow-up were analyzed. *Conclusions*: Considering these long-term results, hypofractionated SIB-VMAT can be considered a viable treatment choice, even for patients with unfavorable conditions.

## 1. Introduction

Adjuvant whole breast radiotherapy (WBR) is an effective treatment for early-stage breast cancer patients who have undergone conservative surgery with adequate margins. This treatment can reduce the relative risk of local recurrence by almost 70% at 5 years [1]. In previous studies, conventional fractionation schemes involve a total dose of 45–50.4 Gy delivered to the whole breast, along with fractionated doses of 1.8–2.0 Gy given 5 days per week. An additional boost dose of 10–12 Gy is given to the tumor bed [2,3]. However, the conventional fractionation treatment regimen takes 5–7 weeks to complete. Some breast cancer patients who undergo conservative surgery do not receive postoperative irradiation, with up to 30–36% of patients foregoing treatment. This may be due to the challenge of adhering to the standard 5–7-week protocol and the higher costs and longer waiting lists associated with conventional radiotherapy schedules [4,5].

Hypofractionation has emerged as a popular alternative in recent years for breast cancer treatment due to its potential to reduce the duration of radiotherapy. There are radiobiological justifications for the use of hypofractionation in breast cancer. The fractionation sensitivity of tissues is measured by the α/β ratio in terms of the linear quadratic (LQ) iso-effect formulation, and the smaller this ratio, the higher the sensitivity. The α/β value for breast cancer has been estimated to be 4 Gy, which is lower than the value for soft tissues, which is approximately 3.5 Gy. These differences in α/β values support the use of hypofractionation, as it allows for a larger dose to be delivered to the tumor bed in each fraction while limiting the overall dose delivered to the surrounding healthy tissues [6]. The similar α/β values for breast cancer and surrounding healthy tissues suggest that high-fraction doses may produce similar radiotherapy sensitivity in both types of tissues in terms of late reactions. However, higher-fraction doses may also be more effective in destroying tumor cells, which is a potential advantage of hypofractionation in breast cancer treatment.

Studies have demonstrated that the recommended α/β ratio for acute radiation reactions in normal tissues such as skin is 10 Gy, whereas the recommended α/β ratio for breast cancer is around 4 Gy, which is comparable to late-reacting normal tissues [7]. Therefore, utilizing hypofractionated treatment for breast cancer can kill more tumor cells than the conventional 2 Gy per fraction and prevent the repopulation of tumor cells during RT.

The findings from several clinical trials suggest that hypofractionated whole-breast irradiation (HF-WBI) is a viable treatment option for women with early-stage breast cancer. These trials show that HF-WBI produces outcomes similar to traditional fractionation in terms of both safety and efficacy [7,8,9]. The latest guidelines from the American Society of Radiation Oncology endorse hypofractionated whole-breast irradiation (HF-WBI) as the preferred treatment choice for early-stage breast cancer. The guidelines recommend techniques such as intensity-modulated radiation therapy (IMRT) and volumetric-modulated arc therapy (VMAT) to ensure appropriate dose distribution, particularly in cases where breast anatomy and volume may pose a challenge. These techniques help deliver higher doses of radiation to the tumor bed while minimizing the dose to surrounding healthy tissues, leading to better treatment outcomes and fewer side effects [10].

In recent years, several large-scale randomized trials with follow-up periods of up to 10 years have demonstrated that using a hypofractionated approach for breast cancer treatment leads to comparable levels of toxicity and outcomes as compared to the traditional fractionation method. These findings support hypofractionation as a safe and effective treatment option for women with early-stage breast cancer [11,12,13].

This study aims to evaluate the use of hypofractionated whole-breast irradiation (HF-WBI) in conjunction with simultaneous integrated boost (SIB) for breast cancer treatment. This treatment involves delivering a total dose of 48.06 Gy to the whole breast with an additional boost of 0.33 Gy per day into the tumor cavity over a total of 18 fractions, reaching a total of 54 Gy. This study utilizes techniques such as intensity-modulated radiation therapy (IMRT), volumetric-modulated arc therapy (VMAT), and hybrid methods to deliver the radiation dose. The goal is to determine the benefits of hypofractionated radiation therapy for breast cancer patients and to further address questions about using this treatment approach.

## 2. Materials and Methods

In this study, 125 eligible patients who underwent conservative surgery at our institute between 2014 and 2016 were assessed. None of the patients had any major violations. All patients met the following inclusion criteria: age above 50 years, stage T1-2 N0 disease, and a central axis dose of 95% to 105%.

Table 1 presents the characteristics of the patients, tumor, surgery, and radiotherapy (RT). 

### 2.1. Radiotherapy

The radiation therapy was started within 4 weeks of the surgical procedure or the last dose of chemotherapy. In patients with stage T1-2No breast cancer, the radiotherapy area was determined as the whole breast and a boost to the tumor site.

The hypofractionated treatment for the breast consisted of 18 sessions, each delivering a dose of 2.67 Gy, resulting in a total dose of 48.06 Gy. A simultaneous integrated boost method was used to deliver a boost of 3.0 Gy per day to the tumor bed. The patients received treatment once per day, five days per week. To avoid exceeding the prescribed treatment time, missed radiation sessions were made up on weekends. No significant changes were made to the treatment process throughout the course of treatment.

The patients were positioned in the supine position on a carbon breast board with the ipsilateral arm up and head turned to the opposite side, and a CT simulation was performed with a 3 mm slice thickness. The Eclipse version 13 treatment planning system was used to generate IMRT plans, which were delivered using a Varian TrueBeam STx 2.0 (Varian Medical. Systems, Palo Alto, ABD). The dose was calculated using an anisotropic analytical algorithm. The target volume dose was prescribed according to the recommendations of the International Commission on Radiation Units and Measurement (ICRU) Reports 50 and 62. In patients with stage T1-2N0 breast cancer, the radiotherapy area was determined as the whole breast and boost to the tumor site. The planning target volume (PTV) for the whole breast and tumor bed volume were defined according to the recommendations of the breast cancer atlas for radiation therapy planning consensus definitions of the Radiation Therapy Oncology Group (RTOG). The determination of targets and organs at risk, as well as dose coverage, was conducted in adherence to the guidelines set by the International Commission on Radiation Units and Measurements. Plans were made using dose–volume histograms. Daily online cone-beam CT (CBCT) was used for treatment verification. Table 2 provides structure/target volumes and dosimetric characteristics.

### 2.2. Patient Evaluation

The study analyzed the skin’s reaction to radiation therapy, and the severity of dermatitis was assessed using the RTOG grading system. The highest grade of dermatitis observed during treatment was used for analysis. The time from the first radiation fraction to the point when dermatitis reached grade 1 or 2 was also recorded and analyzed.

The patients were evaluated for toxicity by the treating physician every week during the radiation treatment. Subsequently, follow-up visits were scheduled every 3 months for up to 2 years, every 6 months for up to 5 years, and then once per year. The physician evaluated toxicities and classified them using the RTOG scale [14].

In this trial, all patients were provided with information and given the validated Turkish version of the Functional Assessment of Cancer Therapy-Breast (FACT-B) survey. The FACT-B survey consists of two modules: the FACT-General (FACT-G), which assesses the overall quality of life (QOL) in cancer patients, and a breast cancer-specific module consisting of nine items. The FACT-G module consists of 27 items divided into 4 domains. A higher score on the survey indicates a better QOL. The patients completed the first survey within 1 week before starting radiation therapy, the second survey 3 months after completing radiation therapy, and the last survey 5 years after finishing radiation therapy.

The cosmetic outcomes of the treatment were evaluated using a 4-point Likert scale, with scores assigned based on the appearance of the breast [15]. A medium or low score on this scale was considered a “failure” regarding the cosmetic outcome.

### 2.3. Statistical Methods

The data were organized and analyzed using the statistical software SPSS 18.0 (IBM SPSS Statistics for Windows, Version 18.0. New York, IBM Corp, Armonk, NY, USA). Descriptive statistics such as mean, standard deviation, and percentage were used to summarize the patient’s basic information, quality of life, and IDA scores. *t*-tests were performed to compare the quality of life and RTOG evaluation scores between the baseline and follow-up surveys. A general linear model and the multivariable analysis technique were used to identify the significant factors and measure their multivariate effects. A *p*-value of less than 0.05 was considered statistically significant in the analyses.

## 3. Results

The study had a median/mean follow-up period of 72 months, ranging from 60 to 96 months, and during this time, no recurrence was observed either locally or in the surrounding region. The overall survival and event-free survival rates were both 100%. The average breast volume was 810 cc, ranging from 500 to 1800 cc, and the mean volume of the boost region was 132 cc, ranging from 54 to 210 cc, according to the data presented in Table 2.

During radiotherapy, 23% of the patients had grade 1 skin toxicity, while 13% had grade 2. After 3 months, 13% had grade 1 skin toxicity (grade 2:6%); after 5 years, only 6% had grade 1 skin toxicity. Before radiation therapy, 10% of patients reported breast pain, with grade 1 pain in 10% of cases. After 3 months of radiation therapy, 17% of patients had breast pain (grade 1:17%); after 5 years, 5% had breast pain (grade 1:5%). Additionally, 10% of patients experienced breast edema before radiation therapy, which increased to 17% after 3 months and decreased to 5% after 5 years. Please refer to Table 3 for more information.

At the final follow-up appointment, the majority of patients (90.4%) had an excellent/good cosmetic outcome, while a small percentage (9.6%) had a good/fair cosmetic outcome (as shown in Table 4).

Over the 5-year follow-up period, FACT-B total scores improved and varied significantly (*p* = 0.04) (see Table 5).

In terms of breast volume, physical well-being scores were worse in patients with breast volumes over 700 ccs, although this difference was not statistically significant (*p* = 0.057). The general QoL data were similar to others.

No statistically significant differences were found when patients were divided into those aged ≥ 70 years and those under 70. However, worse trends were observed in patients aged ≥ 70 years (*p* = 0.059).

According to the results of the study, there was no statistically significant relationship between the following factors and toxicity: body mass index (BMI), breast volume, the presence of additional health conditions (HT, DM), chemotherapy treatment, and radiotherapy techniques.

## 4. Discussion

In this study, we aimed to evaluate the acute and late skin changes, as well as the quality of life, of patients with breast cancer who underwent adjuvant whole-breast radiotherapy after conservative surgery. Previous studies have suggested that the fraction size of radiotherapy has a greater impact on late effects than acute effects [16,17,18]. Hypofractionated radiotherapy has been found to have reduced acute toxic effects due to the higher value of α/β for acute skin reactions. In a large multicenter cohort analysis, Jagsi et al. found that hypofractionation not only improves convenience but may also reduce acute pain, fatigue, and dermatitis. However, their cohort only included patients who received hypofractionated radiotherapy to the whole breast without a boost to the tumor bed [19]. In contrast, this study included patients who received whole breast radiotherapy with a concurrent tumor bed boost using a simultaneous integrated boost technique (SIB).

The study by Shaitelman et al. found lower acute skin effects and good quality of life for patients treated with hypofractionated breast radiotherapy compared to conventional fractionation [20]. In their randomized trial, patients were allocated to either HF-WBI (42.56 Gy in 16 fractions of WBI) or CF-WBI (50.00 Gy in 25 fractions of WBI), and tumor bed boosts were added as necessary. After three years of treatment, the results of hypofractionation and conventional fractionation were comparable, and tumor bed boost, chemotherapy, and larger breast size did not appear to be significant barriers to hypofractionated WBI [21]. These findings are similar to the results of the current study, despite differences in the boost method.

The study by Dantonio et al. suggested that using a hypofractionated schedule for radiation therapy may be beneficial in several ways. For example, the low occurrence of skin atrophy and fibrosis indicates that this approach may be less harmful to the skin than other methods. This study also found that patients were generally satisfied with the appearance of their skin after treatment, indicating a good cosmetic outcome. Finally, this study reported a high grade of local and distant disease control, meaning that the hypofractionated schedule effectively controlled the growth and spread of the treated disease. Overall, these findings suggest that a hypofractionated schedule may be a promising approach for radiation therapy [22].

Assessing intrinsic radiosensitivity in tissues may not fully account for external factors such as hypoxia, immune cell infiltrates, and other microenvironmental influences that can potentially impact the tissue’s response to radiation. Studies have demonstrated that comorbidities such as diabetes mellitus, hypertension, obesity, and autoimmune diseases increase the rates and severity of RT toxicity [23,24,25]. Weng et al. discovered that women with bra cup sizes of D or larger had lower scores on the FACT-B trial outcome index (E = −2.04; *p* = 0.03), but BMI did not differ by treatment arm (P interaction = 0.69) [26]. However, our trial found no association between breast volume and FACT scores. The present study found no significant relationship between BMI, breast volume, additional health conditions (comorbidities), chemotherapy treatment, and toxicity and the outcome evaluated. It is important to note that the absence of a statistically significant relationship does not necessarily mean that there is no relationship, as there may be limitations in the study’s design or methodology that prevent the detection of a significant relationship. Further research may be necessary to verify these findings.

VMAT-based techniques are advantageous for treating complex targets due to their ability to manage hot and cold spots, maintain target coverage, and preserve critical organs. De Rosa et al. and Venjakob et al. showed that hypofractionated VMAT-based techniques can be well-tolerated with minimal side effects and satisfactory cosmetic outcomes and can also offer improved protection of critical organs compared to traditional 3D-CRT. Although our study did not assess toxicity through dosimetric analysis, we did not find any correlation between the radiotherapy technique and toxicity [27,28].

The study by Wang et al. reported that 65% of seventy-one patients had increased breast asymmetry one year after radiation therapy. This study found that breast volume and supraclavicular nodal irradiation were the most significant factors related to changes in breast asymmetry *(p* < 0.05) [29]. However, our study found no correlation between breast volume and cosmesis.

The study revealed that using hypofractionated breast radiotherapy led to a positive outcome with no significant increase in acute or late side effects, ultimately resulting in a better quality of life for patients. This suggests that hypofractionation may be a safe treatment option for patients with unfavorable conditions, such as a high BMI, large breast volume, or comorbidities, who require radiation therapy. This information can assist healthcare providers and patients in making informed decisions about treatment options. This study also provides information on short-term and long-term toxic effects, cosmetic outcomes, and quality of life for patients undergoing hypofractionated breast radiotherapy with the whole-breast and SIB technique.

## Figures and Tables

**Table 1:** Patient characteristics.

*No. of Patients*		*125*
*Age (range)*		mean 63 (58–84)
*BMI*		mean 29.1(18–33)
*Diabetes Mellitus*		25 (20%)
*Hypertension*		59 (47.2%)
*Tumor location*	Right	60 (48%)
	Left	65 (52%)
*Tumor quadrant*	Upper outer	62 (49.6%)
	Upper inner	23 (28.75%)
	Lower outer	12 (9.6%)
	Lower inner	3 (2.4%)
*Histology*	IDC	90 (72%)
	ILC	19 (15.2%)
	Intraductal	16 (12.8%)
*T stage*	Tis	16 (12.8%)
	T1	67 (53.6%)
	T2	42 (33.6%)
*Grade*	G1	29 (23.2%)
	G2	66 (52.8%)
	G3	30 (24%)
*ER status*	+	98 (78.4%)
	−	27 (21.6%)
*Systemic therapy*	Chemotherapy	59 (47.2%)
	Hormonotherapy	98 (78.4%)
	Trastuzumab	24 (19.2%)
*Radiotherapy technique*		
	IMRT alone	91 (72.8%)
	VMAT alone	12 (9.6%)
	IMRT + VMAT hybrid	22 (17.6%)

**Table 2:** Structure/target volumes and dosimetric characteristics.

*Structure/Target volumes/Dosimetric characteristics*	Results
*Breast Volume*	
* Mean (range) cc*	810 (500–1800)
*Tumor bed volume*	
* Mean (range) cc*	132 (54–210)
*PTV whole breast*	
* Mean dose (Gy)*	49.1
*Mean Maximum dose (Gy)*	49.5
*D98 (Gy)*	45.1
*V95 (%)*	92.5
*V105 (%)*	28.7
*PTV boost*	
*Mean dose (Gy)*	53.2
*Mean Maximum dose (Gy)*	55.1
*D98*	51.7
*V95 (%)*	92.7
*V105 (%)*	0.4

**Table 3:** Toxicity outcomes.

Toxicity	RTOG Toxicity Score	Therapy (*n*) Acute	3 Months (n) Acute	5 Years (*n*) Late
Skin				
	G1	23	13	6
	G2	13	6	-
	G3	-	-	-
Pain				
	G1	10	17	5
Edema				
	G1	15	14	3

**Table 4:** Cosmetic outcome.

Cosmetic Outcome	Before Radiotherapy *n* (%)	3 Months *n* (%)	5 Years *n* (%)
Excellent/good	112 (89.6)	110 (88)	113 (90.4)
Fair/poor	13 (10.4)	15 (12)	12 (9.6)

**Table 5:** FACT (Functional Assessment of Cancer Therapy) scores.

Item	Mean +/− SD			*p*
	Baseline	3 Months	5 Years	
*PHYSICAL WELL-BEING*	20.3 +/− 5.2	20.7 +/− 3.5	22.4 +/− 3.6	0.04
*SOCIAL/FAMILY WELL-BEING*	14.0 +/− 5.3	14.1 +/− 5.0	16.2 +/− 3.8	0.03
*EMOTIONAL WELL-BEING*	18.3 +/− 3.5	19.1 +/− 3.6	19.9 +/− 3.2	0.04
*FUNCTIONAL WELL-BEING*	17.0 +/− 4.7	17.4 +/− 3.6	18.3 +/− 3.1	0.04
*ADDITIONAL CONCERNS*	22.9 +/− 4.4	19.1 +/− 4.4	17.1 +/− 3.8	0.04
*FACT-G Score*	69.6 +/− 11.3	70.6 +/− 10.1	75.0 +/− 10.3	0.02
*FACT-B Score*	136.2 +/− 3.6	137.1 +/− 3.7	137.7 +/− 3.1	0.04

## Data Availability

All data generated or analyzed during this study are included in this article. Further enquiries can be directed to the corresponding authors.

## References

[1] Early Breast Cancer Trialists’ Collaborative Group (EBCTCG) (2005). Effects of radiotherapy and differences in the extent of surgery for early breast cancer on local recurrence and 15-year survival: An overview of the randomized trials. Lancet.

[2] Bartelink H., Horiot J.C., Poortmans P., Struikmans H., Bogaert W., Barillot I., Fourquet A., Borger J., Jager J., Hoogenraad W. (2001). Recurrence rates after treatment of breast cancer with standard radiotherapy with or without additional radiation. N. Engl. J. Med..

[3] Bartelink H., Horiot J.C., Poortmans P., Struikmans H., Bogaert W., Barillot I., Fourquet A., Borger J., Jager J., Hoogenraad W. (2007). Impact of the higher radiation dose of local control and survival in breast-conserving therapy of early breast cancer: 10-year results of the randomized boost versus no boost EORTC 22881-10882 trial. J. Clin. Oncol..

[4] Lawenda B.D., Riffenburgh R.H., Fukuda L., Johnstone P.A. (1999). Invasive breast cancer in women age 75 and older (abstract). Radiology.

[5] Virnig B., Habermann E., AlRaie W., Gerber B. (2007). Increased use of breast-conserving surgery: Preferred treatment or failure to provide adequate treatment?. Breast Cancer.

[6] Yarnold J., Haviland J. (2010). Pushing the limits of hypofractionation for adjuvant whole breast radiotherapy. Breast.

[7] Owen J.R., Ashton A., Bliss J.M., Homewood J., Harper C., Hanson J., Yarnold J.R. (2006). Effect of radiotherapy fraction size on tumor control in patient with early-stage breast cancer after local tumor excision: Long-term results of a randomized trial. Lancet Oncol..

[8] The START Trialists’ Group (2008). The UK standardization of breast radiotherapy (START) trial A of radiotherapy hypofractionation for treatment of early breast cancer: A randomized trial. Lancet Oncol..

[9] The START Trialists’ Group (2008). The UK standardization of breast radiotherapy (START) trial B of radiotherapy hypofractionation for treatment of early breast cancer: A randomized trial. Lancet Oncol..

[10] Smith B.D., Bellon J.R., Blitzblau R., Freedman G., Haffty B., Hahn C., Halberg F., Hoffman K., Horst K., Moran J. (2018). Radiation therapy for the whole breast: Executive summary of an American Society for Radiation Oncology (ASTRO) evidence-based guideline. Pract. Radiat. Oncol..

[11] Yarnold J., Ashton A., Bliss J., Homewood J., Harper C., Hanson J., Yarnold J.R., Bentzen S., Owen R. (2005). Fractionation sensitivity and dose response of late adverse effects in the breast after radiotherapy for early breast cancer: Long-term results of a randomized trial. Radiother. Oncol..

[12] Whelan T.J., Pignol J.P., Levine M.N., Julian J.A., MacKenzie R., Parpia S., Shelley W., Grimard L., Bowen J., Lukka H. (2010). Long-term results of hypofractionated radiation therapy for breast cancer. N. Engl. J. Med..

[13] Haviland J.S., Owen J.R., Dewar J.A., Agrawal R.K., Barrett J. (2013). START Trialists’ Group. The UK Standardisation of Breast Radiotherapy (START) trials of radiotherapy hypofractionation for treatment of early breast cancer: 10-year follow-up results of two randomized controlled trials. Lancet. Oncol..

[14] Cox J.D., Stetz J., Pajak T.F. (1995). Toxicity criteria of the Radiation Therapy Oncology Group (RTOG) and the European Organization for Research and Treatment of Cancer (EORTC). Int. J. Radiat. Oncol. Biol. Phys..

[15] Sert F., Ozsaran Z., Eser E., Alanyalı S.D., Haydaroglu A., Aras A. (2007). Functional Assessment for patients with breast cancer Questionnaires-v.4. Functional Assessment for patients with breast cancer. J. Breast Cancer.

[16] Aaronson N.K., Bartelink H., van Dongen J.A., van Dam F.S.A.M. (1988). Evaluation of breast-conserving therapy: Clinical, methodological and psychosocial perspectives. Eur. J. Surg. Oncol..

[17] Joiner M., van der Kogel A. (2009). Basic Clinical Radiobiology.

[18] Hall E.J., Giaccia A.J. (2006). Radiobiology for the Radiologist.

[19] Jagsi R., Griffith K.A., Boike T.P., Walker E., Nurushev T., Grills I.S., Pierce L.J. (2015). Differences in the acute toxic effects of breast radiotherapy by fractionation schedule: A comparative analysis of physician-assessed and patient-reported outcomes in a large multicenter cohort. JAMA Oncol..

[20] Shaitelman S.F., Schlembach P.J., Arzu I., Shaitelman S.F., Schlembach P.J., Arzu I., Ballo M., Bloom E.S., Buchholz D., Smith B.D. (2015). Acute and short-term toxic effects of conventionally fractionated vs. hypofractionated whole-breast irradiation a randomized clinical trial. JAMA Oncol..

[21] Shaitelman S.F., Lei X., Thompson A., Schlembach P., Bloom E.S., Arzu I.Y., Buchholz D., Chronowski G., Dvorak T., Grade E. (2018). Three-year outcomes with hypofractionated versus conventionally fractionated whole-breast irradiation: Results of a randomized, noninferiority clinical trial. J. Clin. Oncol..

[22] Dantonio L., Cozzi S., Tunesi S., Brambilla M., Masini L., Pisani C., Gambaro G., Magnani C., Krengli M. (2016). Hypofractionated radiation therapy for breast cancer: Long-term results in a series of 85 patients. Tumori.

[23] El-Nachef L., Al-Choboq J., Restier-Verlet J., Granzotto A., Ferlazzo M., Bouchet A., Bourguignon M. (2021). Human Radiosensitivity and Radiosusceptibility: What Are the Differences?. Int. J. Mol. Sci..

[24] Sun C., Zhang M., Qiao Q., Wang Y. (2021). Integrating Intrinsic Radiosensitivity and Immune Status for Predicting Benefits of Radiotherapy in Head and Neck Squamous Cell Carcinoma. Med. Sci. Monit..

[25] Mészáros N., Farkas G., Székely G., Kocsis Z.S., Kelemen P.B., Fodor J., Polgár C., Jurányi Z. (2019). Progressive breast fibrosis caused by extreme radiosensitivity: Oncocytogenetic diagnosis and treatment by reconstructive flap surgery. Cancer Rep. (Hoboken).

[26] Weng J.K., Lei X., Schlembach P., Bloom E.S., Shaitelman S.F., Arzu I.Y., Chronowski G., Dvorak T., Grade E., Hoffman K. (2021). Five-Year Longitudinal Analysis of Patient-Reported Outcomes and Cosmesis in a Randomized Trial of Conventionally Fractionated Versus Hypofractionated Whole-Breast Irradiation. Int. J. Radiat. Oncol. Biol. Phys..

[27] De Rose F., Fogliata A., Franceschini D., Iftode C., Navarria P., Comito T., Franzese C., Fernandes B., Masci G., Torrisi R. (2018). Hypofractionation with a simultaneous boost in breast cancer patients receiving adjuvant chemotherapy: A prospective evaluation of a case series and review of the literature. Breast.

[28] Venjakob A., Oertel M., Alexander Hering D., Moustakis C. (2021). Uwe Haverkamp & Hans Theodor Eich. Hybrid volumetric modulated arc therapy for hypofractionated radiotherapy of breast cancer: A treatment planning study. Strahlenther. Und Onkol..

[29] Wang D., Yang X., He J., Lin J., Henry S., Brown G., Chu L., Godette K.D., Kahn S.T., Liu T. (2020). Impact of Regional Nodal Irradiation and Hypofractionated Whole-Breast Radiation on Long-Term Breast Retraction and Poor Cosmetic Outcome in Breast Cancer Survivors. Clin. Breast Cancer.

